# Assessment of emergency management capabilities for public health emergencies in China

**DOI:** 10.3389/fpubh.2026.1890143

**Published:** 2026-08-12

**Authors:** Minghua Zhou

**Affiliations:** Department of Administration Office, Luzhou People's Hospital, Luzhou, Sichuan, China

**Keywords:** China, emergency management capacity, explanatory factor analysis, public health emergencies, regional disparities

## Abstract

**Objective:**

To analyze emergency management capabilities for public health emergencies in China and provide a basis for decision-making aimed at enhancing these capabilities.

**Methods:**

Based on data from 31 provincial-level administrative regions in China from 2019 to 2023, explanatory factor analysis was used to identify the main factors of the 16 evaluation indicators reflecting emergency management capabilities for public health emergencies, calculate the factor scores for each year, and rank them.

**Results:**

Four main factors were extracted from the 16 evaluation indicators, and the cumulative variance explained after rotation was 85.172%, indicating a satisfactory extraction results. The factor scoring model is F = 0.397 × F_1_ + 0.266 × F_2_ + 0.171 × F_3_ + 0.165 × F_4_, where F_1_ is designated as the multi-stakeholder coordination factor, F_2_ is designated as the socioeconomic operation factor, F_3_ is designated as the healthcare security factor, and F_4_ is designated as the infrastructure construction factor. In 2023, Beijing, Henan and Sichuan ranked in the top three with comprehensive factor scores of 0.805, 0.785, and 0.720, respectively, indicating that these three regions have strong emergency management capabilities. In 2023, Ningxia, Hainan, and Tibet ranked in the bottom three with comprehensive factor scores, with scores of −0.891, −1.002, and −1.423, respectively, indicating that these three regions have relatively weak emergency management capabilities. From 2019 to 2023, emergency management capability rankings remained relatively stable in most regions, though significant changes were observed in regions such as Tianjin, Fujian, Guizhou, and Jilin.

**Conclusion:**

Emergency management capacity in China is primarily influenced by the multi-stakeholder coordination factor, socioeconomic operation factor and healthcare security factor. The development of emergency management capacity varies across different regions in China. Regions such as Shandong, Zhejiang, Jiangsu and Henan have relatively strong emergency management capabilities, while regions such as Ningxia, Qinghai, and Tibet have relatively weak emergency management capabilities.

## Introduction

In recent years, the increasing frequency of public health emergencies worldwide has become serious issue (1). The 2020 eruption of the global pandemic, COVID-19, has had a severe impact on public health systems and socioeconomic development worldwide. Although the COVID-19 pandemic has caused a huge impact in the short term, it has comprehensively reshaped the health emergency system in the long term and put forward new requirements and challenges for the resilience and modern governance level of various countries to deal with major public health emergencies (2). These public health emergencies pose severe threats to the safety of people's lives and property, socioeconomic stability, and national security. They also expose the current shortcomings and inadequacies in emergency management capabilities. Therefore, there is an urgent need to optimize and enhance the current public health emergency management capabilities (3). “Public health emergency management capabilities” refers to the comprehensive capabilities of the government and social system to minimize health damage and social loss through prevention and preparation; monitoring and early warning; rapid response; efficient disposal; and recovery and reconstruction of the whole chain operation in public health emergencies. This capability is no longer a single skill, but the result of the interaction between multiple social subjects, medical resources, economic development and infrastructure. It provides the normal operation of economic and social order and maintains the life safety and health of the people through the multi-system synergy of government leadership, department linkage, and social participation. In March 2026, the “Outline of the 15th 5-Year Plan for National Economic and Social Development of the People's Republic of China” called for improvements to the public health system and the establishment and strengthening of systems for preventing and controlling of infectious diseases, responding to emergencies, providing medical treatment, and supplying materials. To effectively safeguard the lives and property of the people and promote stable socioeconomic development, it is essential to enhance emergency management capabilities for public health emergencies comprehensively (4). Therefore, scientifically and accurately evaluating emergency management capabilities and identifying key improvement factors is crucial for enhancing emergency management capacity.

Currently, studies on emergency management capabilities for public health emergencies are primarily divided into three areas. First, the evaluation and improvement of emergency management systems, mainly including the development and validation of an evaluation tool for public health emergency management plans (5), the construction of an evaluation indicator system for the emergency management capabilities of major infectious diseases in urban communities (6), and a comparison of the financing of health systems of six European member states in the context of multiple economic crises and pandemic outbreaks (7). Second, a comprehensive assessment of emergency management capabilities, which primarily includes an evaluation of emergency response capabilities and obstacle analysis in China (8), an evaluation of emergency management capabilities for urban public health emergencies in Henan Province (9), and an evaluation of capabilities for rural public health emergencies management in Jiangsu Province (10). Third, an analysis of public health emergency preparedness, response, and recovery capabilities, mainly including Pakistan's infodemic management and public health emergency preparedness capabilities (11), leadership training for public health emergency response (12), building public health emergency preparedness, response, and recovery capabilities through disaster citizen science (13), and the core capabilities of public health personnel at disease prevention and control centers in northeast China (14). Currently, studies on emergency management capabilities for public health emergencies in China primarily focus on research methods such as the entropy-weighted method, the analytic hierarchy process, and the Theil index. There are deficiencies in eliminating the information redundancy of multiple evaluation indexes and retaining key information. These studies cover the entire process of emergency management, such as emergency preparedness, response, and recovery capabilities. There are deficiencies in the construction of comprehensive capacity evaluation indicators of multi social subjects, medical resources, economic development, and infrastructure. The research areas primarily involve emergency capability assessments in provinces such as Henan and Jiangsu, and there are deficiencies in the evaluation of the whole region's collaborative construction of emergency management capabilities. On the whole, the current research on the emergency management capabilities of public health emergencies at the level of 31 provincial administrative regions in China is still insufficient. In the evaluation index system, a relatively comprehensive evaluation index system including multi-stakeholder coordination, socioeconomic operation, healthcare security, and infrastructure construction is established. In the research scope, the comparative analysis is made at the level of 31 provincial administrative regions in China. In the research method, factor analysis is used to mine the common driving factors behind variables that cannot be noted directly so as to enhance the interpretability of the results. These are the principal contents that need to be solved urgently in the current research. Accurate and scientific assessments of emergency management capabilities are crucial for identifying and addressing weaknesses, which is significant for safeguarding public health and safety and for preventing and mitigating major risks in the public health sector.

To scientifically and accurately assess emergency management capabilities, this study used explanatory factor analysis to identify the main factors among the 16 evaluation indicators reflecting emergency management capabilities for public health emergencies in China, calculated the factor scores for each year from 2019 to 2023, and ranked them, and explored pathways for enhancing these capabilities in China.

## Methods

### Data sources

Data on emergency management capabilities for public health emergencies in China were obtained from the China Health Statistics Yearbook and the China Statistical Yearbook from 2020 to 2024. The scope of the study is the data on emergency management capabilities for public health emergencies in the 31 provincial-level administrative regions (excluding Hong Kong, Macau, and Taiwan) in mainland China from 2019 to 2023.

### Indicators

Public health emergencies are characterized by their sudden onset, widespread impact, contagious nature, severity, and complexity. These emergencies place high demands on social resources and information, require complex emergency response measures, necessitate coordinated efforts across multiple departments, and have far-reaching social and economic implications (15). The evaluation of public health emergency management capacity is a complex system evaluation, involving management systems, medical and health security, economic and livelihood operations, emergency preparedness, and other factors. The management system needs multi-stakeholder coordination, and the coordination mechanism of government responsibility, department coordination, and social participation is used to ensure the unity of government orders and the efficient allocation of resources. In the multi-stakeholder coordination, the grassroots organizations and trade union organizations are the most basic governance units, which play an important role in organizing and serving the masses; social organizations can play a role in integrating social resources; colleges and universities are important institutions serving the people's livelihood; they form the multi-subject of social governance together. Medical and health security is the core defense line, which directly undertakes the functions of “prevention, control, and governance,” and is the professional support ability of emergency response. The number of medical and health institutions, beds, and health technicians are the core resources of the medical and health system, which determine the health service ability of emergency treatment. Basic medical insurance can reduce the burden of medical expenses of insured personnel and provide important financial guarantees for medical treatment. The economy and livelihood operation needs to maintain the stability of social and economic order, actively promote the resumption of work and production, and prevent economic collapse. General public budget revenue is the financial basis for the government to perform its functions, and general public budget expenditure is an important means of macro-control to jointly ensure the improvement of people's livelihood and social equity. Per capita disposable income and per capita consumption expenditure can measure the living standard of residents and are an important basis for improving people's livelihood policies. Infrastructure construction is the specific performance of the level and emergency conversion ability of places, logistics, data, information, and facilities, which determines the emergency preparedness response speed and the upper limit of carrying capacity. Roads, automobiles, the Internet, radio, and television together constitute the basic support for the logistics, human flow, and data flow of society. In summary, a management system determines command efficiency and responsibility implementation; medical and health security determine the speed of life saving and transmission blocking; economic and livelihood operations determine social resilience and order maintenance; emergency preparedness determines the speed of response and resource supply (16), and they jointly shape the overall emergency response capacity through system coordination, resource flow, risk buffer, and emergency conversion mechanisms.

Based on the characteristics of public health emergencies, relevant research literature (17), and the objectivity and availability of data for each evaluation indicator, 16 evaluation indicators were selected to construct a system for evaluating emergency management capabilities in China. These indicators fall into four categories: multi-stakeholder coordination, healthcare security, socioeconomic operation, and infrastructure construction (Table 1).

**Table 1:** Evaluation indicators system for emergency management capabilities in China.

No.	Categories	Indicators	Description of the indicators
1	Multi-stakeholder coordination	Number of social organizations	Number of social organizations at year-end
2		Number of grassroots organizations	Number of grassroots organizations at year-end
3		Number of colleges and universities	Number of general and vocational colleges and universities at year-end
4		Number of grassroots trade union organizations	Number of grassroots trade union organizations at year-end
5	Healthcare security	Number of healthcare institutions	Number of healthcare institutions per 1,000 population
6		Number of beds in healthcare institutions	Number of beds in healthcare institutions per 1,000 population
7		Number of healthcare technicians	Number of healthcare technicians per 1,000 population
8		Basic medical insurance participation rate	Number of basic medical insurance enrollees/total population × 100%
9	Socioeconomic operation	Per capita disposable income	Per capita disposable income of residents for the year
10		Per capita consumption expenditure	Per capita consumption expenditure of residents for the year
11		General public budget revenue	General public budget revenue per 1,000 population
12		General public budget expenditures	General public budget expenditures per 1,000 population
13	Infrastructure construction	Total road mileage	Road mileage per square kilometer
14		Number of privately owned cars	Number of privately owned cars per capita GDP
15		Internet broadband subscribers	Internet broadband subscribers per capita GDP
16		Number of cable TV subscribers	Number of cable TV subscribers per 1,000 population

### Research methods

Explanatory factor analysis was used to identify the main factors of the 16 evaluation indicators reflecting emergency management capabilities for public health emergencies, calculate the factor scores for each year from 2019 to 2023, and rank them.

The basic principle of explanatory factor analysis is grouping variables based on the strength of their correlations to ensure high correlations among variables within the same group and low correlations between groups (18). This allows each group of variables to represent a basic structure, from which common factors reflecting the interdependencies among the variables can be identified. These common factors are then used to simplify the analysis of complex problems. Explanatory factor analysis was conducted using IBM SPSS Statistics 24.0, and the basic steps are as follows (19):

Step 1: standardize the original data.

Step 2: use the KMO and Bartlett's test to assess the data. If the results show a KMO value > 0.6 and *P* < 0.05, the data is deemed suitable for explanatory factor analysis.

Step 3: calculate the variance explanation table and analyze the eigenvalues, variance contribution, cumulative variance contribution, and other metrics.

Step 4: extract the main factors, calculate the communality of factor loadings, and name and interpret the main factors.

Step 5: calculate the factor scores. Based on each factor's contribution to the total variance, determine each factor's weight according to its proportion of the cumulative variance contribution across several factors, compute the comprehensive factor scores, and rank them.

## Results

### KMO and Bartlett's test

Take the data from 31 provincial-level administrative regions in China in 2023, for example, to perform the calculation. The results show that the KMO value is 0.707 (larger than 0.6), and the Bartlett's sphericity test statistic is 593.553 with *P* < 0.001 (*P* < 0.05). These results indicate a significant correlation among the variables, making them suitable for explanatory factor analysis.

### Table of variance contribution rates

Based on the eigenvalues exceeding 1 in the variance contribution rates, four main factors were extracted. After rotation, these four main factors accounted for 33.850%, 22.678%, 14.593%, and 14.051% of the variance contribution rates, respectively, for a cumulative variance contribution rate of 85.172%, indicating that these four main factors effectively represent the emergency management capabilities of China (Table 2).

**Table 2:** Table of variance contribution rates.

Indicators	Initial eigenvalues			Contribution rate of variance before rotation			Contribution rate of variance after rotation		
	Eigenvalues	variance contribution rate	Cumulative variance contribution rate	Eigenvalues	variance contribution rate	Cumulative variance contribution rate	Eigenvalues	variance contribution rate	Cumulative variance contribution rate
1	6.562	41.015	41.015	6.562	41.015	41.015	5.416	33.850	33.850
2	4.660	29.124	70.139	4.660	29.124	70.139	3.628	22.678	56.528
3	1.396	8.724	78.863	1.396	8.724	78.863	2.335	14.593	71.121
4	1.009	6.309	85.172	1.009	6.309	85.172	2.248	14.051	85.172
5	0.665	4.158	89.330	–	–	–	–	–	–
6	0.479	2.994	92.323	–	–	–	–	–	–
7	0.337	2.108	94.432	–	–	–	–	–	–
8	0.301	1.881	96.313	–	–	–	–	–	–
9	0.175	1.094	97.407	–	–	–	–	–	–
10	0.161	1.009	98.416	–	–	–	–	–	–
11	0.092	0.572	98.988	–	–	–	–	–	–
12	0.065	0.407	99.395	–	–	–	–	–	–
13	0.052	0.328	99.723	–	–	–	–	–	–
14	0.031	0.193	99.916	–	–	–	–	–	–
15	0.009	0.056	99.972	–	–	–	–	–	–
16	0.004	0.028	100.000	–	–	–	–	–	–

### Factor loading coefficients after rotation

After extracting four main factors, calculating the degree of commonality of the evaluation indicators revealed maximum and minimum values of 0.975 and 0.663, respectively, indicating satisfactory overall factor extraction. Based on the factor loadings after rotation, the first main factor, F_1_, has significant loadings on indicators such as the number of colleges and universities and the number of grassroots trade union organizations, which primarily reflects the level of multi-stakeholder coordination; it is named the multi-stakeholder coordination factor. The second main factor, F_2_, has significant loadings on indicators such as the general public budget income and per capita disposable income, which primarily reflects the level of socioeconomic operation; it is named the socioeconomic operation factor. The third main factor, F_3_, has significant loadings on indicators such as the number of beds in healthcare institutions and the basic medical insurance participation rate, which primarily reflects the level of healthcare security; it is named the healthcare security factor. The fourth main factor, F_4_, has significant loadings on indicators such as the total road mileage and the number of cable TV subscribers, which primarily reflects the level of infrastructure construction; it is named the infrastructure construction factor (Table 3).

**Table 3:** Factor loading coefficients after rotation.

Indicators	Factor loading coefficients				Degree of commonality
	F_1_	F_2_	F_3_	F_4_	
Number of social organizations	0.895	−0.005	−0.154	0.242	0.883
Number of grassroots organizations	0.841	−0.236	0.383	−0.107	0.921
Number of colleges and universities	0.912	0.078	0.104	0.293	0.935
Number of grassroots trade union organizations	0.927	−0.024	−0.026	0.207	0.903
Number of healthcare institutions	−0.075	−0.313	0.148	−0.839	0.829
Number of beds in healthcare institutions	0.261	−0.038	0.820	−0.029	0.743
Number of healthcare technicians	−0.201	0.861	0.130	−0.083	0.805
Basic medical insurance participation rate	0.109	−0.458	0.690	0.072	0.703
Per capita disposable income	0.086	0.805	−0.503	0.259	0.975
Per capita consumption expenditure	0.105	0.765	−0.499	0.340	0.961
General public budget revenue	−0.121	0.826	−0.444	0.132	0.911
General public budget expenditures	−0.444	0.248	−0.355	−0.649	0.806
Total road mileage	0.386	0.162	−0.049	0.697	0.663
Number of privately owned cars	0.892	−0.165	0.204	0.024	0.865
Internet broadband subscribers	0.880	−0.231	0.306	0.097	0.931
Number of cable TV subscribers	−0.377	0.658	0.045	0.464	0.793

### Factor score coefficient matrix

**Figure 1:**
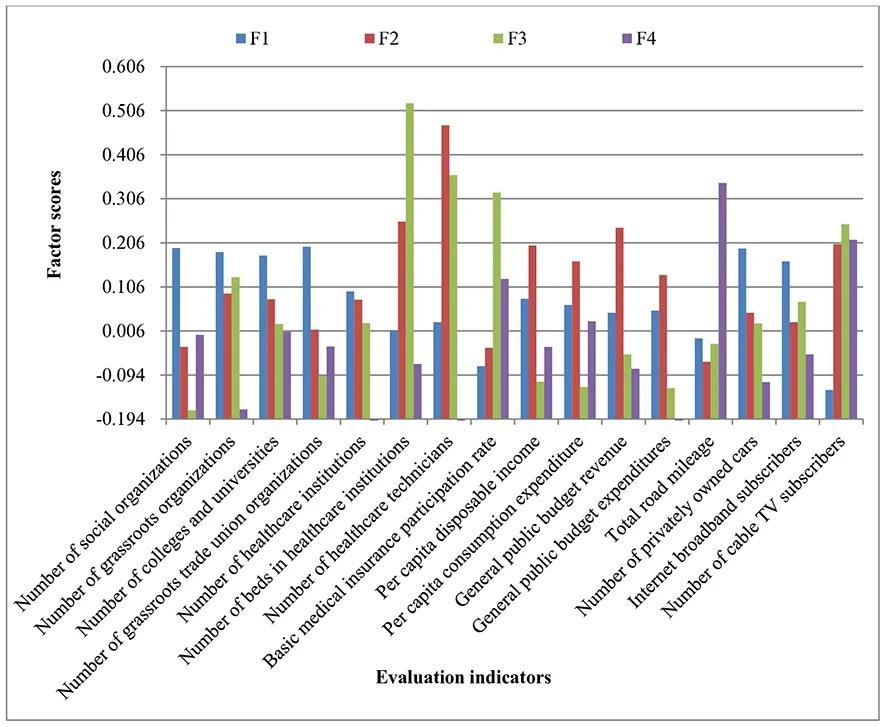
Factor score coefficient matrix.

The scores for each main factor can be obtained based on the factor component score coefficient matrix. Then, the comprehensive factor score is then calculated using the variance contribution rates of each main factor as weights, according to the formula: F = 0.397 × F_1_ + 0.266 × F_2_ + 0.171 × F_3_ + 0.165 × F_4_, which enables calculation of the comprehensive factor scores and their ranking (Figure 1).

### Comprehensive factor scores and rankings by region

**Figure 2:**
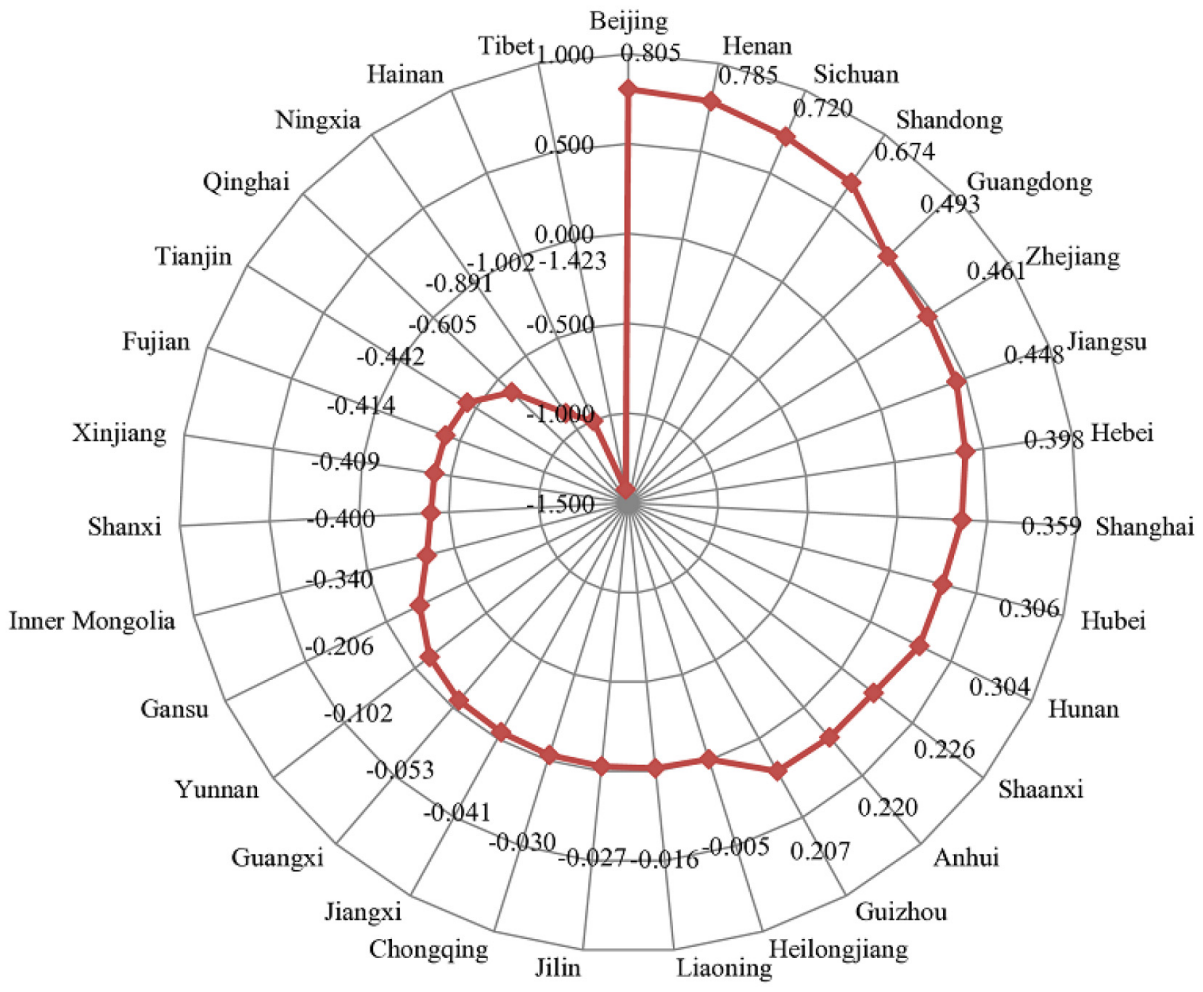
Comprehensive factor scores and rankings by region.

An analysis of the comprehensive factor scores for each region reveals that Beijing, Henan and Sichuan ranked in the top three, with scores of 0.805, 0.785, and 0.720, respectively, indicating that these three regions have strong emergency management capabilities. Ningxia, Hainan, and Tibet ranked in the bottom three, with comprehensive factor scores of −0.891, −1.002, and −1.423, respectively, indicating that these three regions have relatively poor emergency management capabilities. 14 regions, including Beijing and Henan, have comprehensive factor scores greater than 0, while the remaining 17 regions have scores less than 0, indicating significant disparities in emergency management capabilities across regions (Figure 2).

Beijing is the center of politics and international exchanges and is in the leading position in terms of universities, social organizations, economic development, medical resources, and infrastructure construction in China. The management system attaches great importance to construction and has high emergency efficiency, which leads to a better comprehensive score. Henan and Sichuan have a large population and good urbanization, which are the core bearing areas of the central and western regions, respectively, with convenient transportation and a strong rescue force, thus having a good comprehensive score. The development of medical resources and infrastructure in Hainan is relatively late, and the integration and development of emergency management capabilities will take more time. Ningxia and Tibet have poor economic development and slow infrastructure construction, which leads to relatively weak emergency management capacity. Compared with Sichuan, which is also located in the western region, Chongqing's medical capacity and infrastructure construction are relatively weak, resulting in a lower comprehensive score.

### Main factor extraction in 2019–2023

According to the above calculation steps, calculate the main factor extraction from 2019 to 2023. The cumulative variance contribution rate is above 78%, indicating that the extraction is good. Four main factors were extracted in 2019, 2021, and 2023, which were named the multi-stakeholder coordination factor, socioeconomic operation factor, healthcare security factor, and infrastructure construction factor. There are three main factors extracted in 2020 and 2022, which are named the multi-stakeholder coordination factor, socioeconomic operation factor, and healthcare security factor (Table 4).

**Table 4:** Main factor extraction in 2019–2023.

Year	Cumulative variance contribution rate	Number of main factors	Naming of main factors
2019	83.387	4	Multi-stakeholder coordination factor, socioeconomic operation factor, infrastructure construction factor, healthcare security factor
2020	78.125	3	Multi-stakeholder coordination factor, socioeconomic operation factor, healthcare security factor
2021	85.147	4	Multi-stakeholder coordination factor, socioeconomic operation factor, healthcare security factor, infrastructure construction factor
2022	78.779	3	Multi-stakeholder coordination factor, socioeconomic operation factor, healthcare security factor
2023	85.172	4	Multi-stakeholder coordination factor, socioeconomic operation factor, healthcare security factor, infrastructure construction factor

### Comprehensive factor scores and rankings by region, 2019–2023

From 2019 to 2023, Shandong, Zhejiang, Jiangsu and Henan ranked in the top 10 for comprehensive factor scores, while Ningxia, Qinghai, and Tibet ranked in the bottom five, indicating uneven development of emergency management capabilities across regions. From 2019 to 2023, regions that experienced significant changes in their rankings include Tianjin (down 12 places), Fujian (down 10 places), Jiangsu, Liaoning and Inner Mongolia (down 4 places), Guizhou (up 9 places), Jilin (up 8 places), Henan (up 6 places), Jiangxi and Gansu (up 5 places), Sichuan and Anhui (up 4 places); the rankings of emergency management capabilities in the remaining 19 regions is relatively stable (Table 5).

**Table 5:** Comprehensive factor scores and rankings by region, 2019–2023.

Regions	2019		2020		2021		2022		2023	
	F	Ranking	F	Ranking	F	Ranking	F	Ranking	F	Ranking
Beijing	1.030	1	0.640	6	0.818	1	0.341	11	0.805	1
Henan	0.441	8	0.512	7	0.688	3	0.738	2	0.785	2
Sichuan	0.565	7	0.205	10	0.752	2	0.513	7	0.720	3
Shandong	0.672	5	0.720	5	0.663	4	0.708	3	0.674	4
Guangdong	0.844	2	1.346	1	0.252	12	0.539	5	0.493	5
Zhejiang	0.780	4	0.972	3	0.488	7	0.490	8	0.461	6
Jiangsu	0.823	3	1.020	2	0.608	6	0.758	1	0.448	7
Hebei	0.302	10	0.365	8	0.256	11	0.137	14	0.398	8
Shanghai	0.649	6	0.904	4	0.372	9	0.328	12	0.359	9
Hubei	0.162	11	0.134	12	0.274	10	0.528	6	0.306	10
Hunan	0.368	9	0.114	13	0.408	8	0.376	9	0.304	11
Shaanxi	0.130	13	−0.120	17	0.618	5	0.080	17	0.226	12
Anhui	−0.181	17	0.108	14	0.151	14	0.594	4	0.220	13
Guizhou	−0.283	23	−0.388	23	0.157	13	0.320	13	0.207	14
Heilongjiang	−0.021	14	−0.368	22	−0.031	18	−0.107	21	−0.005	15
Liaoning	0.138	12	−0.050	15	0.076	15	0.035	18	−0.016	16
Jilin	−0.360	25	−0.586	26	−0.023	17	−0.223	23	−0.027	17
Chongqing	−0.215	20	−0.158	18	−0.016	16	0.369	10	−0.030	18
Jiangxi	−0.303	24	−0.118	16	−0.144	20	0.082	16	−0.041	19
Guangxi	−0.262	22	−0.158	19	−0.126	19	0.084	15	−0.053	20
Yunnan	−0.203	18	−0.203	20	−0.148	21	−0.024	19	−0.102	21
Gansu	−0.466	27	−0.685	29	−0.363	24	−0.478	25	−0.206	22
Inner Mongolia	−0.211	19	−0.445	24	−0.325	23	−0.649	28	−0.340	23
Shanxi	−0.232	21	−0.303	21	−0.281	22	−0.413	24	−0.400	24
Xinjiang	−0.402	26	−0.589	27	−0.539	27	−0.643	27	−0.409	25
Fujian	−0.151	16	0.217	9	−0.363	25	−0.061	20	−0.414	26
Tianjin	−0.071	15	0.174	11	−0.417	26	−0.155	22	−0.442	27
Qinghai	−0.717	29	−0.948	30	−0.692	28	−0.979	30	−0.605	28
Ningxia	−0.705	28	−0.603	28	−0.872	30	−0.677	29	−0.891	29
Hainan	−0.783	30	−0.470	25	−0.819	29	−0.574	26	−1.002	30
Tibet	−1.336	31	−1.241	31	−1.424	31	−2.037	31	−1.423	31

The comprehensive scores of Tianjin and Fujian declined significantly, which was due to the impact of multiple outbreaks of COVID-19. The regional static management was higher than average level in China, which had a significant impact on economic development, commercial circulation, and residents' lives. Although the government took active measures to ensure social stability and promote economic recovery, the results have been mediocre. The comprehensive scores of Guizhou and Jilin increased significantly because they were less affected by the COVID-19 epidemic, and society, economy, and transportation were in normal operation, which was contrary to the situation in other regions that were greatly affected by the epidemic's closure and control, which led to the decline of the ranking. In this case, these regions have developed steadily, and the ranking has gradually improved.

## Discussion

The emergency management capacity for public health emergencies in China is primarily related to factors of multi-stakeholder coordination, socioeconomic operations and healthcare security. Hospitals, schools, and trade union organizations have played an active role in emergency management, fostering collaboration among multiple stakeholders and enhancing emergency response capabilities (20). They are not only the first responders to emergencies, but also an important bridge connecting the government emergency system and the public. Public health emergency requires the participation of the whole society, collective efforts and efficient coordination in order to effectively deal with public health emergency. In the socioeconomic security, growth in both per capita disposable income and per capita consumer expenditure show that the social and economic development is better, the people's income growth, in the face of public health emergency, can have a better ability to pay. With the development of social economy, the public's health awareness has gradually increased, and their willingness to pay for healthcare has grown. This contributes to an overall improvement in emergency response capabilities (21). China has continuously strengthened its healthcare system, promoted the development of medical and public health resources, and conducted regular emergency drills and training. These efforts enhance emergency response capabilities and practical skills in medical treatment (22). Medical resources, such as the number of beds in healthcare institutions and the number of healthcare professionals, have continued to grow, and integrating routine and emergency care has provided greater flexibility for emergency treatment. The number of basic medical insurance participation rate has remained stable, which has lightened the financial burden of medical treatment for the general public. In addition, emergency management capacity and infrastructure construction also have great relevance; especially digital technology provides solutions for the whole life cycle of public health emergency, which has the potential to significantly improve management efficiency and effectiveness (23). Therefore, it is essential to continuously improve the emergency management system for public health emergencies, enhance practical capabilities through medical treatment training and drills, fully leverage the active role of grassroots organizations such as hospitals, schools, and labor unions, and foster collaboration among multiple stakeholders (24). It is also necessary to further strengthen socioeconomic development, improve the multi-tiered social security system, enhance public health education and awareness, and promote the gradual development of emergency management capabilities (25).

From 2019 to 2023, the ranking of emergency management capabilities in most regions of China has remained relatively stable, indicating that the development of such capabilities is a long-term process. While all regions are actively working to improve their public health emergency management systems and increase investment in public health, building such capabilities requires sustained and stable investment to yield positive results (26). There are regional disparities exist in the development of emergency management capabilities. In general, regions with larger populations and stronger economies tend to have better emergency management capabilities, while regions with relatively smaller populations and weaker economies tend to have weaker emergency management capabilities (27). For a long time, social resources have been allocated based on population density. Regions with high population density tend to have better-developed public health services, as well as well-established emergency response capabilities, including monitoring, early warning, and medical treatment (28). Regions with stronger economic development allocate more fiscal revenue to constructing emergency management systems, thereby enhancing the public's ability to pay for medical care and strengthening emergency management capabilities (29). Effective emergency management is not only based on population and economy but also depends on management systems, emergency preparedness, and effective use of resources. For example, the economic level of Hebei is at a relatively low level in the eastern region, but Hebei has implemented the mechanism innovation of the emergency management list system and promulgated the legal innovation of the Regulations on the Transfer of Flood Control and Risk Avoidance Personnel in Hebei Province. At the same time, Hebei has strengthened the Beijing-Tianjin-Hebei joint drill and information sharing and material coordination mechanism, and its emergency management capability is at a better level. From 2019 to 2023, regions that experienced significant changes in their rankings included Tianjin (down 12 places), Fujian (down 10 places), and other regions, which is due to the impact of the outbreak of COVID-19, which has a significant impact on economic development, commercial circulation, and residents' lives. Emergency response capabilities in these regions continue to improve, but at a relatively slow pace, resulting in their rankings declining as other regions overtake them (30). Emergency management capabilities in these regions are inadequate, particularly due to the weakness of grassroots emergency response forces and insufficient initial response capacity, while the complexity of the risks poses new challenges to these capabilities. Therefore, all regions should develop their emergency management capabilities according to local conditions; strengthen the emergency management systems, staffing, monitoring, and early warning mechanisms, and material support infrastructure at the grassroots level; as well as enhance their ability to respond to all kinds of public health emergencies in a timely manner. Regions with better emergency management capabilities should improve the integration mechanism of government coordination with social subjects to participate in emergency management, strengthen the construction of emergency teams, improve the efficiency of emergency rescue, and quickly carry out emergency rescue in surrounding areas, so as to improve the overall efficiency of emergency management. Regions with poor emergency management capabilities should increase financial investment in public emergency, strengthen infrastructure construction, conduct regular emergency training and drills, improve the quality and level of public health emergency response, and narrow the differences in emergency management capabilities caused by differences in economic development.

From 2019 to 2023, Shandong, Zhejiang, Jiangsu and Henan ranked in the top 10 for their comprehensive factor scores, indicating strong emergency management capabilities in these regions. In addition to factors such as the region's robust economic development and well-developed infrastructure, these regions have also achieved significant results in areas such as innovation of emergency management systems, grassroots capacity building, and public participation. These results promote comprehensive development of emergency management capabilities (31). Shandong has pioneered the establishment of a system for appointing safety directors and implementing dual leadership. The province has also established and operationalized a provincial emergency command center, vigorously developed an aviation rescue system, consistently prioritized prevention, and focused efforts on transforming the work safety governance model toward preemptive prevention. In addition to establishing a comprehensive emergency response organizational structure and command mechanism, Jiangsu prioritized developing diverse emergency rescue capabilities. The province boasts a large number of highly capable emergency rescue teams and has fostered specialized civilian rescue groups to facilitate the orderly participation of social forces (32). This has significantly enhanced its emergency management capabilities and provided a solid safety foundation for high-quality development. Henan, Zhejiang and other regions have established and refined its emergency command system, creating a framework of division of labor and collaboration characterized by “unified coordination during emergencies and specialized responsibilities during peacetime.” These regions have achieved full coverage of emergency response plans at the grassroots level, with townships establishing dedicated emergency rescue teams to enhance local emergency management capabilities (33). These regions have built a modern, professional, and intelligent emergency management system through institutional innovation, technological support, grassroots empowerment, legal safeguards, and public participation (34). Therefore, these regions should further improve the quality of emergency prevention and control, establish and improve the emergency system with the participation of multiple social subjects, and enhance the emergency response capacity and efficiency of each subject in responding to health emergencies. It is also necessary to give full play to the advantages of better emergency management capabilities, summarize appropriate systems and experiences for nationwide promotion, and promote the sustainable development of emergency management capabilities in other regions.

From 2019 to 2023, Ningxia, Qinghai, and Tibet ranked in the bottom five for their comprehensive factor scores, indicating relatively weak emergency management capabilities in these regions. While these regions have made some progress in developing these capabilities, their location in western China, characterized by vast, sparsely populated areas and underdeveloped economies, combined with an incomplete emergency management system, has resulted in their overall weakness. These regions have a weak historical foundation and have long suffered from insufficient investment, resulting in a late start in developing infrastructure such as emergency rescue equipment, communication systems, and shelters, a shortage of specialized rescue personnel, and slow progress in building emergency management capabilities (35). These regions have poor transportation infrastructure and long travel distances to public health services. This limits the coverage of medical emergency services in rural areas and hinders the effective transmission of emergency information and coordination mechanisms. Consequently, there are significant shortcomings in coverage and emergency response efficiency in remote areas, which makes it difficult to provide a rapid response and sustained support in complex disaster scenarios (36). These regions also face challenges such as the inadequate development of emergency response teams, a lack of systematic training for grassroots teams, insufficient emergency drills and practical experience, and limited participation from civil society (37). This makes it difficult to foster a collaborative effort among multiple stakeholders to build emergency management capabilities. The functional simulation exercise of the National Public Health Emergency Operations Center in the African region helped to understand the existing response systems and procedures and the role in emergency management, which showed that it was effective to organize all parties to participate in the exercise (38). Therefore, these regions should learn from areas with stronger emergency management capabilities, gradually improve their emergency management systems, increase financial investment, gradually improve infrastructure construction (39), and make full use of information technology to achieve monitoring, early warning, and rescue coordination mechanisms (40). It is also necessary to further strengthen the development of emergency response teams, conduct emergency training and drills, and improve the coordination efficiency and overall synergy of public health emergencies.

## Limitations

This study has some limitations. First, although we included as many evaluation indicators as possible, the impact of each indicator on emergency management capacity varies, which inevitably influences the evaluation results. The indicators analyzed mainly measure the availability of resources, which cannot fully reflect the efficiency, service quality, and actual operational capacity in emergency situations. Although we use relative indicators, some absolute indicators may bring structural advantages to regions with larger economies and populations. In future studies, the indicators should include indicators adjusted according to population, region, or economic level and use relative indicators for more accurate assessment. Second, although we use exploratory factor analysis to identify the structure and association between variables, it does not allow drawing causal conclusions about the factors that determine the performance of emergency management. In future studies, the use of panel models, cluster analysis, or machine learning-based methods to determine different readiness for public health emergencies could be explored, suggesting a deeper explanation of the impact mechanism. Third, emergency management capacity is a dynamic, evolving process influenced by factors such as policies and major epidemics, and the explanation of this study is insufficient. Although we included an analysis of the impact of the COVID-19 outbreak, we did not separately analyze the impact of the crisis on the evolution of investment priorities, health system structures, and preparedness strategies. In future studies, we can focus on analyzing the differences between the pre-epidemic, epidemic, and post-epidemic periods.

## Conclusions

To improve emergency management capabilities for public health emergencies in China, we established an evaluation index system with 16 indicators, including four aspects: multi-stakeholder coordination, socioeconomic operation, healthcare security, and infrastructure construction. We used exploratory factor analysis to identify the main factors, calculated the factor scores for each year, and ranked them. The study found that emergency management capacity in China is primarily influenced by the multi-stakeholder coordination, socioeconomic operation and healthcare security factors. It shows that the capability to respond to public health emergency needs multi-dimensional interaction, so that administrative capacity, economic development, and social infrastructure can contribute to the preparation and response to complex crises.

Factor analysis is effective in eliminating the information redundancy of multiple evaluation indicators and retaining key information, which can be applied to build a comprehensive analysis framework for cross-regional comparison, and can effectively evaluate the specific situation of health emergency management capacity in different regions.

From 2019 to 2023, the ranking of emergency management capability in most regions is relatively stable, and the ranking changes greatly in Tianjin, Fujian, Guizhou, Jilin and other regions. The development of emergency management capability in different regions of China is uneven. Based on the actual situation of each region, the research results can support the formulation of customized development strategies to improve the regional emergency management capability. We suggest that it is essential to continuously improve the emergency management system for public health emergencies, enhance practical capabilities through training and drills in medical treatment, and fully leverage the active role of grassroots organizations, such as hospitals, schools, and trade union organizations. It is also necessary to further strengthen socioeconomic development, improve the multi-tiered social security system, and promote the gradual development of emergency management capabilities. Regions with better emergency management capabilities should improve the integration mechanism of government coordination with social subjects to participate in emergency management and improve the efficiency of emergency rescue. Regions with poor emergency management capabilities should increase financial investment in public emergencies, strengthen infrastructure construction, and make full use of information technology to achieve monitoring, early warning, and rescue coordination mechanisms.

The research model used in this study can be adjusted and used in the evaluation of public health emergency management capacity in other countries facing similar regional imbalances, and can also be interpreted appropriately by combining panel models, cluster analysis, and other methods.

## Data Availability

The original contributions presented in the study are included in the article/Supplementary material, further inquiries can be directed to the corresponding author.
